# [Corrigendum] Effect of antioxidants coenzyme Q10 and β-carotene on the cytotoxicity of vemurafenib against human malignant melanoma

**DOI:** 10.3892/ol.2026.15778

**Published:** 2026-07-23

**Authors:** Changkun Hu, Yuan Huang, Peixiao Luo, Yixin Yang

Oncol Lett 21: 208, 2021; DOI: 10.3892/ol.2021.12469

Following the publication of the above paper, an interested reader drew to the authors’ attention that, regarding the cell migration and invasion assay data shown in Fig. 3A on p. 6, the 1 μM, 5 μM and 10 μM (β-carotene) data panels, and the 1 μM (β-carotene)+PLX, 5 μM+PTX and 10 μM+PTX data panels, contained a number of overlapping sections, such that data which were intended to show the results of six different experiments had apparently been derived from two original sources.

After consulting their original data, the authors have realized that the data in this figure were inadvertently assembled incorrectly. To rectify this issue, the authors have submitted a revised version of Fig. 3 featuring replacement data for Fig. 3A [which also entailed an updated analytical approach that transitioned from cell counting to the quantification of the migration cell area (%), thereby providing a more precise and objective representation of the results], and this is shown on the next page. The authors wish to highlight that the results obtained were broadly similar to those included in the published paper; however, some changes are necessary concerning the description of these results in the article. Based on their refined analysis, the relevant data have been updated: The migration cell areas are now shown as 15.5% and 11.5% for 5 and 10 µM β-carotene alone, respectively, compared with 34.3% in the control group. Therefore, the text featured in the “*β-carotene inhibits cell invasion, but alleviates the inhibitory effect of PLX on cell invasion*” subsection of the Results (on p. 6–7) should now read as follows (changes are highlighted in bold): “Since it was reported that β-carotene inhibited lung metastasis of murine melanoma *in vivo* (53) and inhibited migration and invasion of human hepatocarcinoma cells *in vitro* (65), based on these findings the present study examined the effects of β-carotene on the invasive ability of A2058 human melanoma cells and on the inhibitory effect of PLX4032 on cell invasion using a Matrigel-coated Transwell cell invasion assay. β-carotene alone at 5 and 10 µM significantly decreased A2058 cell invasion across the cell-permeable microporous membrane to 15.5% and 11.5% (migration cell area), respectively, compared with 34.3% in the control group (Fig. 3A and B). Notably, β-carotene alleviated the inhibitory effect of PLX4032 on A2058 melanoma cell invasion (Fig. 3A and B).”

The authors are grateful to the Editor of *Oncology Letters* for allowing them this opportunity to publish a Corrigendum, and all the authors agree with its publication; moreover, they apologize for any inconvenience caused to the readership.

## Figures and Tables

**Figure 3.**
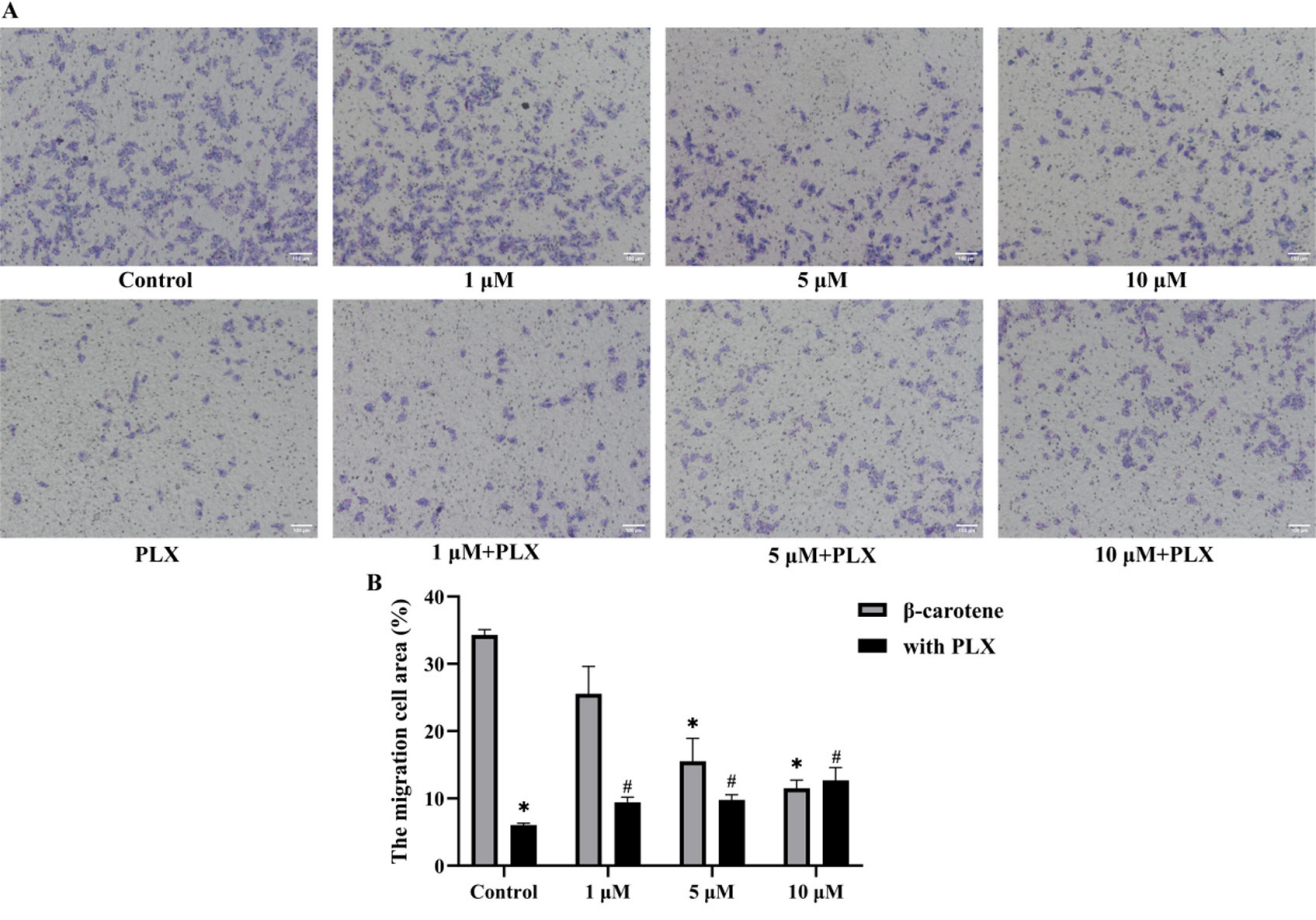
β-carotene inhibits cell invasion and alleviates the inhibitory effect of PLX on cell invasion. (A) Representative microscopic images of migrated A2058 cells in migration/invasion assays demonstrated the inhibitory effect of β-carotene alone, and PLX together with β-carotene. (B) Migrated cell numbers in the control group and different treatment groups. *P<0.05, control group vs. treatment group; #P<0.05, PLX alone group vs. combined treatment groups (PLX and β-carotene group). CoQ10, coenzyme Q10; PLX, vemurafenib.

